# Conjunctival congestion: a novel clinical sign in older children with Tetralogy of Fallot

**DOI:** 10.15190/d.2022.13

**Published:** 2022-09-30

**Authors:** Arun Prasad, Pradeep Kumar, Amit Raj, Yankappa Nayak

**Affiliations:** ^1^Department of Pediatrics, All India Institute of Medical Sciences, Patna, Bihar, India; ^2^Department of Ophthalmology, All India Institute of Medical Sciences, Patna, Bihar, India

**Keywords:** Conjunctival congestion, bulbar conjunctiva, cyanosis, chronic hypoxemia, hyperaemia, clinical finding, tetralogy of Fallot.

## Abstract

Tetralogy of Fallot is the most common cyanotic heart disease in children. While doing echocardiographic examination of children with Tetralogy of Fallot, we observed that many older children with this condition had congestion in their bulbar conjunctiva, easily recognizable even from some distance. This observation led us to design and perform a research study in order to find out the presence of conjunctival congestion in children with Tetralogy of Fallot. 85% of children in the ≥ 5-years of age group had conjunctival congestion without any ocular symptom. This novel clinical finding can act as an adjunct clinical sign for recognizing Tetralogy of Fallot in older children.

## INTRODUCTION

**Figure 1:**
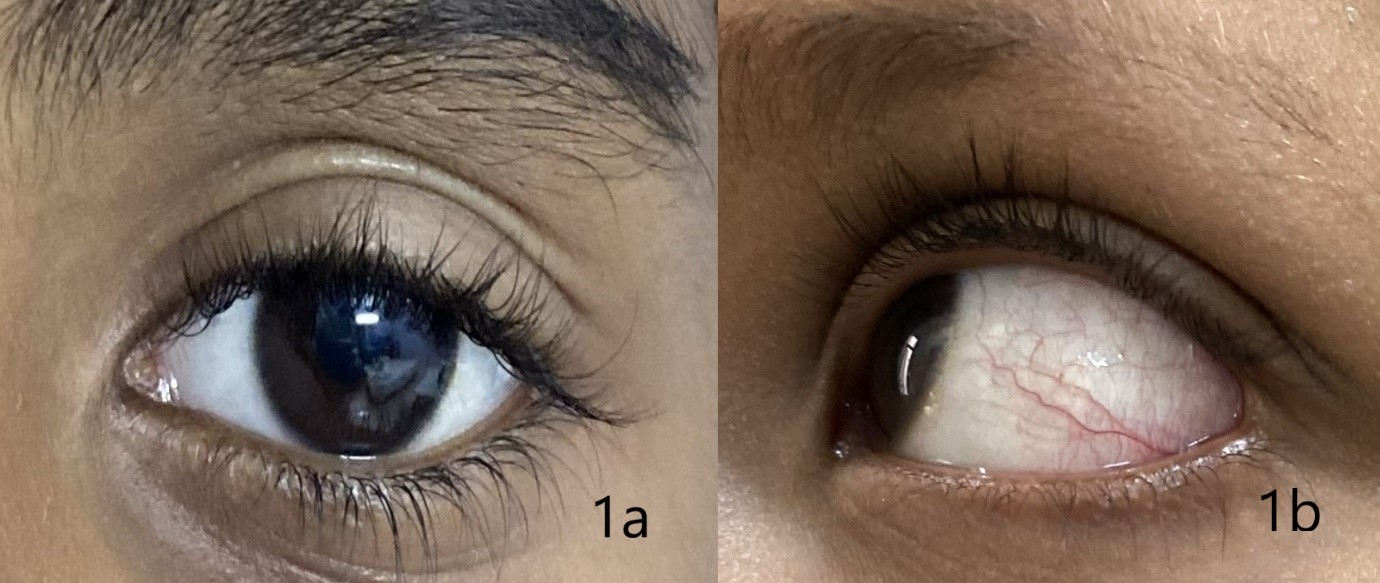
An eight-year-old child with normal eye (1a) and conjunctival congestion in a child of same age with Tetralogy of Fallot (1b)

Tetralogy of Fallot is the most common congenital cyanotic cardiac defect in children^1^. Four components of this structural cardiac anomaly consist of the ventricular septal defect, overriding of aorta, infundibular pulmonary stenosis, and right ventricular hypertrophy^2,3^. This combination of lesions occurs in 3 out of 10,000 live births and accounts for 7-10% of all congenital cardiac malformations^4^. Central cyanosis is a common finding in Tetralogy of Fallot and it is found more consistently in older children, and children with right ventricular outflow obstruction with a higher-pressure gradient between the right ventricle and main pulmonary artery^5^. Anatomical lesions leading to right ventricular outflow obstruction are infundibular stenosis, pulmonary valvular stenosis, and narrow main pulmonary artery with their varying combinations^6^. Sometimes an atretic pulmonary valve is present^7^. Right ventricular outflow obstruction in Tetralogy of Fallot is severe in most of the cases and it leads to the right to left shunt across the ventricular septal defect, causing central cyanosis and chronic hypoxemia^8^. Chronic hypoxemia works as a stimulus for developing polycythemia which, consequently, increases the viscosity of blood. Long-standing polycythemia leads to widespread dilatation and tortuosity of smaller blood vessels in different parts of the body. Polycythemia also leads to other symptoms and complications, such as headache, dizziness, and thromboembolism^9,10^.Observation of dilated and tortuous vessels in the retina has been described in Tetralogy of Fallot in various studies, but conjunctival congestion has not been mentioned in this condition, so far^11^. Clinical features suggestive of the diagnosis of Tetralogy of Fallot are central cyanosis, clubbing and ejection systolic murmur in pulmonary area^12^. While doing echocardiography of more than 250 children with Tetralogy of Fallot, we observed that the older children with this condition have an appearance of congested conjunctiva without any ocular symptom (Figure 1). These observations lead us to do structured research to find any relationship between Tetralogy of Fallot and conjunctival congestion.

## MATERIALS AND METHODS

We conducted an observational study to find out the presence of conjunctival congestion in children with Tetralogy of Fallot, diagnosed by echocardiography. Ethical clearance was obtained from the Ethics Committee of our Institute. We examined 42 children with Tetralogy of Fallot for the presence of congestion in their bulbar conjunctiva. The presence or absence of conjunctival congestion was assessed by clinical examination of the bulbar conjunctiva of both eyes of every child included in this study. Hyperaemia of bulbar conjunctiva was graded as per the ‘Japan Ocular Society’ Scale for the measurement of hyperaemia, by comparing photographs of bulbar conjunctiva with the photograph contained in the said scale^13^.

## RESULTS

Sixteen children in the study were from less than or equal to 5 years age group, whereas 26 children belonged to the age group of more than 5 years old. One out 16 children in the age group of ≤ 5 years old, with history of recurrent cyanotic spell, had grade 2 conjunctival congestion. Out of 26 children, in > 5 years age group, 22 (85%) showed Grade 1 (7/22, 32%) to Grade 2 (15/22, 68%) bulbar conjunctival congestion, as per measurement of hyperaemia by ‘Japan Ocular Society’ Scale. The conjunctival congestion in these children was not associated with any ocular symptom (Table 1).

**Table 1:** Distribution of conjunctival congestion in children with Tetralogy of Fallot

Age group of children with TOF	< 5 years (n = 16)	≥ years (n=26)
Conjunctival congestion Grade 1	00	7
Conjunctival congestion Grade 2	1	15
Total (%)	1 (6%)	22 (85%)

## DISCUSSION

Chronic hypoxemia due to right to left shunt across ventricular septal defect leads to polycythemia with its consequent generalized tortuosity of blood vessels, due to hyperviscosity of blood. It is more obvious in older children and younger children with more severe right ventricular outflow obstruction. We found in our research that conjunctival congestion is a common finding in older children with Tetralogy of Fallot. This ocular finding could be helpful in making a clinical diagnosis of Tetralogy of Fallot, as an adjunct clinical sign. This physiological change of conjunctival congestion as a consequence of polycythemia is expected to be found in children with other chronic cyanotic conditions, such as tricuspid atresia, Eisenmenger syndrome, and Ebstein’s anomaly also^14,15^.

## CONCLUSION

Tetralogy of Fallot is the most common congenital cyanotic heart disease. Clinical diagnosis of this cardiac lesion is made on the basis of typical signs and symptoms, like central cyanosis, clubbing, and ejection systolic murmur in pulmonary area. We have discovered conjunctival congestion as a new clinical sign in older children with Tetralogy of Fallot. This sign could be helpful in making the diagnosis of this structural cardiac disease based on clinical features. However, more comprehensive studies are required to obtain a better evaluation of the findings.
